# Effective manual cleaning as the first step of reprocessing glass probes of a medical device for non-invasive physical plasma therapy

**DOI:** 10.3205/dgkh000486

**Published:** 2024-06-05

**Authors:** Roland Röcker, Benedikt Eggers, Axel Kramer, Matthias B. Stope

**Affiliations:** 1 Dental Center Roland Röcker, Wolfsburg, Germany; 2 Department of Oral, Maxillofacial and Plastic Surgery, University Hospital Bonn, Bonn, Germany; 3 Institute of Hygiene and Environmental Medicine, University Medicine Greifswald, Greifswald, Germany; 4 Physical Plasma Medicine Laboratories, Department of Gynecology and Gynecological Oncology, University Hospital Bonn, Bonn, Germany

**Keywords:** cold plasma, cold atmospheric plasma, noninvasive physical plasma, medical device, cleaning, reprocessing

## Abstract

**Background:**

For reusable devices and device components, effective reprocessing is essential to prevent nosocomial infections.

**Aim:**

The objective of the study was to evaluate manual cleaning as the first step of reprocessing reusable glass probes of a device for generation of non-invasive physical plasma, in accordance with regulations.

**Methods:**

Two glass probes of the device were contaminated with human blood. For manual cleaning, both probes were cleaned with instrument cleaning agent and instrument brushes. Cleaning efficacy was evaluated by total protein measurement in the rinsing solution.

**Results:**

After manual cleaning of the two test glass probes, no protein from the test contamination with human blood could be detected. Neither the different design of the two probes nor the use of a hard or a soft instrument brush demonstrated any difference.

**Conclusion:**

Our data suggest that manual cleaning of glass probes achieves complete removal of organic contaminants. This should enable safe applications in clinical practice.

## Zusammenfassung

**Hintergrund:**

Beim Einsatz wiederverwendbarer Geräte und Gerätekomponenten ist eine effiziente Aufbereitung zur Vermeidung nosokomialer Infektionen unerlässlich.

**Zielsetzung:**

Ziel der Studie war es, die manuelle Reinigung als den ersten Schritt der Aufbereitung von wiederverwendbaren Glassonden eines Geräts zur Erzeugung von nicht-invasivem physikalischem Plasma regelkonform zu evaluieren.

**Methoden:**

Zwei Glassonden des Geräts wurden mit menschlichem Blut kontaminiert. Für die manuelle Reinigung wurden beide Sonden mit Instrumentenreinigungsmittel und Instrumentenbürsten gereinigt. Die Wirksamkeit der Reinigung wurde durch Messung des Gesamtproteins in der Spüllösung bewertet.

**Ergebnisse:**

Nach manueller Reinigung beider Testglassonden konnte kein Protein aus der Kontamination mit menschlichem Blut nachgewiesen werden. Weder das unterschiedliche Design beider Sonden noch die Verwendung einer harten oder einer weichen Instrumentenbürste zeigten Unterschiede.

**Fazit:**

Die Daten weisen darauf hin, dass die manuelle Reinigung von Glassonden die vollständige Entfernung von organischen Verunreinigungen ermöglicht. Das sollte eine sichere Anwendung als ersten Schritt der Aufbereitung in der klinischen Praxis ermöglichen.

## Introduction

Reusable medical devices are contaminated during treatment and must be carefully reprocessed. The first step is thorough cleaning as a precondition for disinfection, because improper cleaning can lead to an accumulation of contamination; especially in terms of pathogens, a-priori cleaning helps avoid the risk of subsequent biofilm formation [1], [2]. The risk of contamination is especially high if the design of the device is complex [3]. In addition to microbial pathogens, organic contaminants can also cause complications. These can be bound to the surfaces of the device through exposure to cleaning solutions, disinfectants or heat. The contaminants can bind disinfectants and neutralize their effectiveness, or physically cover pathogens, thereby protecting them from the disinfection effect. Manual cleaning is therefore essential as the first step in reprocessing, regardless of which reprocessing method is used afterwards. In most cases, significant amounts of tissue residues and biofilms can be removed mechanically using a brush before the actual disinfection [4], [5]. It is assumed that thorough manual cleaning of a medical device reduces the germ load to 20% [6]. 

In Germany, the Medical Devices Act [7], the Medical Devices Operator Ordinance [8] and the recommendation of the Commission of Hospital Hygiene and Infection Prevention and the Federal Institute for Drugs and Medical Devices [9] serve as the legal basis for reprocessing medical devices. The manufacturer’s instructions for the specific reprocessing of the medical device must also be considered.

In this study, manual cleaning of reusable glass probes of a device for generation of non-invasive physical plasma (NIPP) is analyzed by determining the protein load of different glass probes after artificial soiling and manual cleaning using brushes. 

## Method

### Characterization of the device

**Figure 1:**
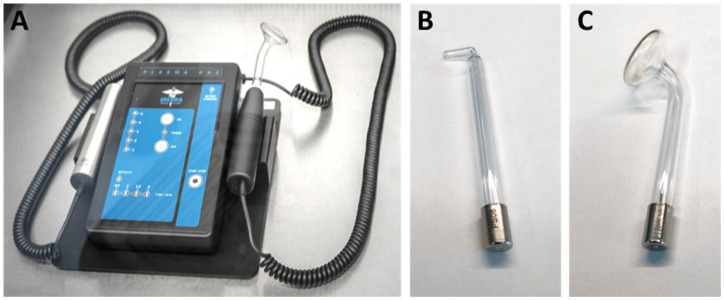
(A) The Plasma One device for generation of non-invasive physical plasma (NIPP); (B) Glass probe PS 08; (C) Glass probe PS 30

The NIPP device Plasma One (Plasma Medical Systems, Nassau, Germany) is a class-IIa medical device with ISO 13485 and ISO 9001 certification and EC certificate of conformity (Figure 1 (Fig. 1)). The device is approved for intraoral application in dental treatment (Figure 1 (Fig. 1)) and can be operated with different glass probes for wound healing, inflammatory and infectious diseases in various areas of human medicine as well as dentistry [10], [11], [12].

### Contamination and cleaning

**Figure 2:**
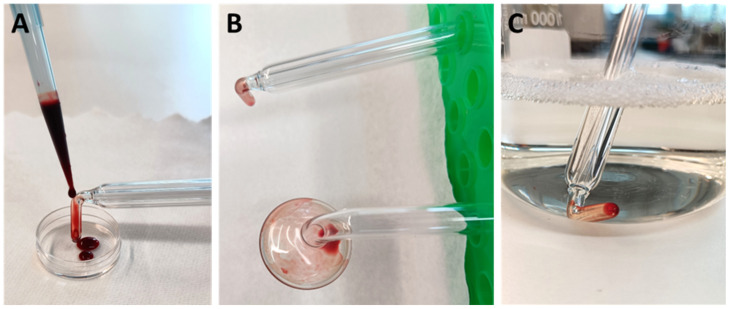
(A) Contamination of the therapeutically active end of the glass probes with 500 µl human whole blood; (B) Air drying of the test blood for 4 h; (C) Soaking of the glass probes in the instrument cleaning agent for 30 min

The DIN EN ISO 15883 standard requires a defined test soiling and soiling method as well as the use of real soiled instruments typical for the operation to check the cleaning performance [13]. The PS 08 and PS 30 probes were selected as test specimens to evaluate manual cleaning. As soil, human blood containing ethylenediaminetetraacetic acid (EDTA) was used. The blood was obtained from an oncological patient in the Department of Gynecology and Gynecologic Oncology at University Hospital Bonn, Germany. The patient was informed and gave her consent (ethics vote of the Ethics Committee of the University Hospital Bonn Az 128/21). Test soiling was performed with 500 µl of human blood, which was applied to the therapeutically active end of each probe using a microliter pipette (Figure 2A (Fig. 2)). The blood was then dried in air for 4 hours at room temperature (Figure 2B (Fig. 2)). Before manual cleaning, the contaminated sections of the probes were immersed in the commercial instrument cleaning agent Neodisher Mediclean Forte (Dr. Weigert, Hamburg, Germany) for 30 min (Figure 2C (Fig. 2)).

**Figure 3:**
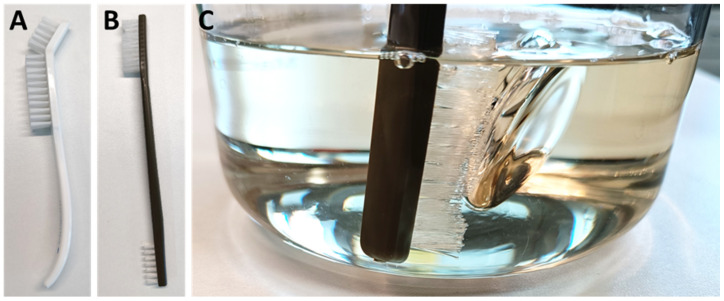
(A) Angled brush with extra-hard nylon bristles (article 8M140001, Medimex, Limburg, Germany); (B) Large (35 mm) and small (25 mm) nylon head brush (article 09098, Keysurgical, Lensahn, Germany); (C) Cleaning the probes by submerging in Neodisher Mediclean Forte instrument cleaning solution.

After soaking the probes in the instrument cleaning agent for 30 min, the probes were cleaned with brush A or B, until no more soiling was visible. Finally, the cleaned probes were thoroughly rinsed with deionized water (Figure 3 (Fig. 3)). 

### Protein analysis of the cleaned glass probes

For proof of cleaning efficacy according to DIN EN ISO 15883, it is required to demonstrate that less than 100 µg total protein is detectable on the test specimen after manual cleaning [13]. For this purpose, the bicinchoninic acid (BCA) method was used, which is robust, sensitive and also able to detect small amounts of protein [14].

**Figure 4:**
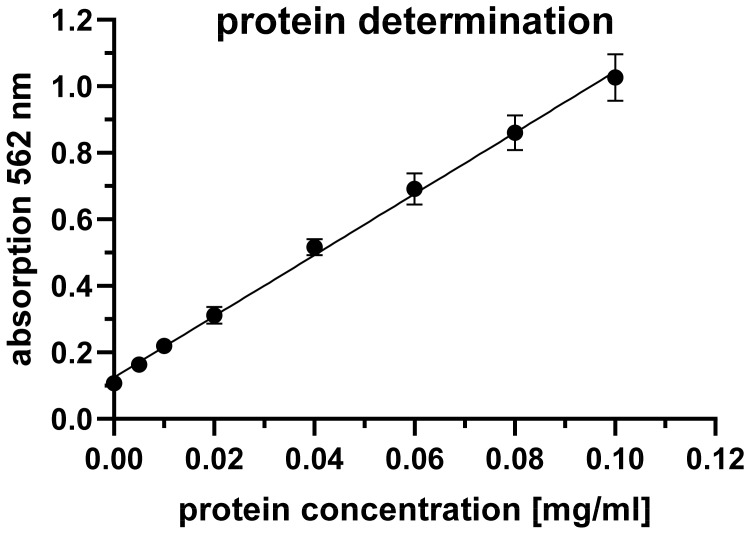
Protein determination using the bicinchoninic acid (BCA) assay. For calibration, 0.0 to 0.1 mg/ml bovine serum albumin (BSA) fraction V was used

For protein analysis, the manually cleaned probes were rinsed 5 times each with 1 ml of 1% sodium dodecyl sulphate (SDS; Carl Roth, Karlsruhe, Germany) solution using a pipette. The probe tip was rinsed five times with the same ml of SDS solution. This enabled the potential protein contamination to be effectively rinsed off the probe without the rinsed proteins being too diluted because too large a volume of rinsing solution was used. The extra-hard angled brush (Medimex) was used four times, the small head twice, and the large head of the soft brush (Keysurgical) four times for cleaning. Subsequently, total protein in 200 µl of the rinsing solution was quantified using the BCA Protein Quantification Kit (article E112-01, Vazyme, Düsseldorf, Germany) according to the manufacturer’s instructions. Protein concentrations were determined using a calibration curve with bovine serum albumin (BSA) fraction V (Carl Roth; Figure 4 (Fig. 4)). The concentration of total protein in the blood sample served as the positive control. 

## Results

Two replicate measurement series of different dilutions (1:160, 1:320, 1:640, 1:1280) showed on average a clear, linear course of absorbance at 562 nm (Table 1 (Tab. 1)). After correction for the dilution factors, this resulted in a very homogeneous value of 12.8 mg/ml total protein at all dilutions.

The measured absorbance (562 nm) averaged 0.109±0.006 (range 0.104–0.124) and corresponded to the zero value of the standard curve. Thus, after taking the total volume into account, no protein was detectable even in 1 ml of 1% SDS rinsing solution after manual cleaning. 

The results between the two probes did not differ. Likewise, the different brush models did not show any qualitative differences. The degree of hardness of the bristles appeared to be irrelevant. The small, 25-mm nylon head of the double brush (Keysurgical) proved to be impractical, as cleaning was more cumbersome and took longer. However, this utility of this characteristic certainly depends on the instrument to be cleaned. For practical implementation, the procedure must be defined in a standard operation procedure including each sub-step to ensure safe, reliable cleaning.

## Discussion

The total protein concentration of 12.8 mg/ml in the control blood appears lower than the clinically defined range of 64.0–83.0 mg/ml [15]. However, this blood parameter is individual and can deviate significantly from the standard range, particularly depending on the disease. In cardiology patients, for example, Ersoy et al. [15] showed total protein values of 43.0–92.0 mg/ml. Parameters that can change after blood collection, such as gas saturation, pH and general handling of the sample, can also lead to significant deviations from normal values ranging, from 28.0–77.0 mg/ml [16], [17]. The total protein value of 12.8 mg/ml determined in the positive control is slightly lower than clinically expected. However, as this was only a qualitative control and the experimental procedure did not reflect standard blood diagnostic laboratory practice, this deviation is negligible.

The limit of 100 µg total protein after manual reprocessing was not exceeded, and reprocessing with the instrument cleaning agent and an instrument brush can be considered sufficient. In a different study, the instrument cleaner was also effective [18].

An important factor in reprocessing efficacy is the instrument material. Glass surfaces are comparatively easy to clean due to their homogeneous surface structure [19]. Glass is also comparable to the gold standard, stainless steel, in terms of biofilm formation and sterilization efficacy [20]. An important advantage of the glass probes tested is probably also the design. The glass body is manufactured without corners, edges, or deep incisions, making cleaning much easier. Manual cleaning by trained personnel is therefore highly likely to enable safe use in clinical applications. 

### Limitation

Although the study was conducted in accordance with the legal regulations, the restriction to BCA protein detection as the only measurement parameter represents a limitation. It would be very interesting for future studies to determine whether the proven effective cleaning of the test specimens also led to sterilization. Elimination of the microbial load could be determined using appropriate microbiological methods. It might then be conceivable that in certain cases (e.g., with certain instrument symmetries and materials) manual cleaning could also be recognized as disinfection and thus full reprocessing of instruments.

## Conclusion

The present study demonstrates the effectiveness of manual cleaning of glass probes using an instrument cleaning agent and instrument cleaning brush. After cleaning, no protein contamination could be detected in the areas contaminated with human blood. The described manual reprocessing of glass probes should therefore enable safe use in clinical practice. Since no protein components could be detected at all, it cannot be ruled out that the manual cleaning may even have led to the complete removal of infectious agents (e.g., viruses, bacteria).

## Notes

### Authors’ ORCID

- Benedikt Eggers: 0000-0002-4274-3801
- Axel Kramer: 0000-0003-4193-2149
- Matthias B. Stope: 0000-0003-4129-8854

### Competing interests

Stope MB is the founder and CEO of 5'-TOP-3' Physical Plasma Medicine.

Röcker R, Eggers B and Kramer A declare that they have no competing interests.

## Figures and Tables

**Table 1:**
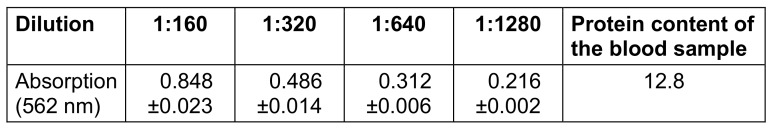
Protein concentration (mg/ml; mean and standard deviation) after serial dilution of the blood sample as positive control
